# COVID-19: cross-immunity of viral epitopes may influence severity of infection and immune response

**DOI:** 10.1038/s41392-021-00490-x

**Published:** 2021-03-01

**Authors:** Junaid Kashir, Khaled AlKattan, Ahmed Yaqinuddin

**Affiliations:** 1 College of Medicine, Alfaisal University, Riyadh, Kingdom of Saudi Arabia; 2 Department of Comparative Medicine, King Faisal Specialist Hospital and Research Center, Riyadh, Kingdom of Saudi Arabia

**Keywords:** Infectious diseases, Antigen processing and presentation

A recent study published in *Science* by Shrock et al.^1^ examined how responses to COVID-19 severity differed amongst patients with different prior viral exposure history, finding notable correlations between both. Such serological profiling provides a window into differential viral responses amongst patients with diverse outcomes, potentially mediating improved therapeutics and vaccines for SARS-CoV-2.^1^

The ongoing global coronavirus disease-2019 (COVID-19) pandemic caused by the novel respiratory coronavirus, severe acute respiratory syndrome coronavirus 2 (SARS-CoV-2), underlies widespread global morbidity, but particularly rapidly overwhelmed medical facilities of Europe and North America. Indeed, only 9% of deaths have occurred in Asia where the outbreak originated, while Europe and North America account for 75% of case fatalities.^2^

Shrock et al.^1^ examined the antibody profiles of 232 COVID-19 patients and 190 pre-COVID-19 era controls, identifying several antibody epitopes shared between other coronaviruses (CoVs) and SARS-CoV-2. COVID-19 patients requiring hospitalization. Such patients were also observed to exhibit stronger and broader antibody responses to SARS-CoV-2, but weaker responses to past infections compared with those who did not need hospitalization.^1^ This perhaps suggests that patients who did not need hospitalization had exhibited a stronger response to previous infections.

Perhaps the stronger the exposure/response to previous CoV infections, the lower the chances that hospitalization was required upon SARS-CoV-2 infection. Indeed, a significantly lower level of antibodies targeting common viruses including rhinoviruses, enteroviruses, and influenza was observed in COVID-19 patients requiring hospitalization compared to those who did not.^1^ However, this could also just be indicative of a lower immune capacity as opposed to acquired immunity through multiple infections. Astoundingly though, in the same study, Shrock et al.^1^ also observed that COVID-19 patients requiring hospitalization exhibited a higher seroprevalence rate for cytomegalovirus (CMV) and herpes simplex virus 1 (HSV-1), indicating that even though exposure to non-CoVs mounted an adequate immune response, this was unable to prevent hospitalization following SARS-CoV-2 infection. Conversely, the hospitalized group of COVID-19 patients exhibited a lower response to CMV and HSV-1 peptides, suggesting a reduced immune capacity.^1^

Seven CoVs have been associated with human disease, mostly amounting to mild respiratory illness, perhaps underlying 15–30% of global common colds. Previous CoV outbreaks have included severe respiratory syndrome (SARS) caused by SARS-CoV, and Middle East respiratory syndrome (MERS) caused by MERS-CoV. Significantly, SARS-CoV, MERS-CoV, and SARS-CoV-2 share significant sequence homology and antigenic epitopes capable of inducing an adaptive immune response.^2^ All CoVs consist of transmembrane trimeric spike (S) glycoproteins, membrane (M) glycoproteins, and transmembrane envelope (E) proteins. The RNA of CoV is bound to nucleocapsid (N) proteins resembling string-on-beads. Due to high levels of homology between these proteins in SARS-CoV, MERS-CoV, and SARS-CoV-2, perhaps prior exposure to one virus could confer partial immunity to another. Thus, perhaps an ‘adaptive immune response’ due to repeated/increased CoV exposure in Asian/Middle Eastern populations underlies low morbidity seen in those regions.^2^

Shrock et al.^1^ indicated that peptides corresponding to the S and N proteins were most reactive in COVID-19 patient sera, but significantly lower in pre-COVID era controls. However, the third most frequently recognized peptide in COVID-19 patient sera was the replicase polyprotein ORF1, recognized to a similar degree in pre-COVID era control sera as well. This was posited to be likely due to cross-reactivity between antibodies elicited by previous CoVs exposure, suggesting that such patterns represented a pre-existing cross-reactive response.^1^ Indeed, mapping highly conserved regions of the S protein between SARS-CoV-2 and other common CoVs, as well as specific regions mapping to individual CoVs, indicated cross-reactivity with SARS-CoV, MERS-CoV, and seasonal CoVs to varying degrees. This phenomenon, perhaps, could potentially be attributed to antibody production induced by other pathogens. Collectively, such data seems to suggest that responses to seasonal CoVs may be able to influence the immune response to SARS-CoV-2.^1^

Taken collectively, the findings of Shrock et al., and increasing numbers of other groups seem to strongly suggest that perhaps previous CoV infection infers an ‘immune response memory’, which could be an explanation as to why locations around the world with a history of CoV infections have yielded a lower morbidity rate.^2^ Indeed, prior viral exposure could provide some protection if cross-reactive neutralizing antibodies or T cell responses are stimulated upon exposure to SARS-CoV-2^1^ (Fig. 1).

**Fig. 1:**
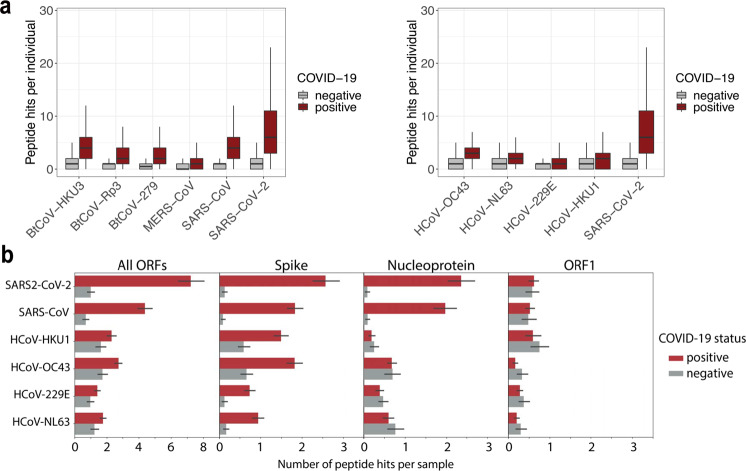
Representative indicators of the findings by Shrock et al.,^1^ suggesting significant overlap between SARS-CoV-2 proteins with other coronaviruses in human patients. **a** Box plots illustrating the number of peptide hits from the indicated coronaviruses in confirmed COVID-19 patients and pre-COVID-19 era controls (negative for SARS-CoV-2). Cross-reactivity toward SARS-CoV-2 peptides was observed in pre-COVID-19 era samples, while COVID-19 patients also exhibited cross-reactivity with other common hCoVs, SARS-CoV, and MERS-CoV. **b** Bar graphs depicting the average number of peptides derived from SARS-CoV-2, SARS-CoV, and each of the four most common hCoVs significantly enriched per sample following IgG immunoprecipitation in the experiments of Shrock et al.^1^ The ORF1 region of SARS-CoV-2 exhibited greatest comparative matches between patients diagnosed with COVID-19 versus those that tested negative, indicating a potentially large degree of overlap between previous CoV and SARS-CoV-2 infection, whereby previous CoV perhaps reduced the severity of SARS-CoV-2 infection. Figure adapted from Shrock et al.,^1^ with permission

Such potential mechanisms whereby patients are rendered with levels of ‘antibody memory’ against various prior viral infections could thus be utilized in both therapeutic and vaccinology applications. One such demonstrative example is that of intravenous immunoglobulin (IVIG) administration, which through formation of antigen–antibody complexes and their neutralization, mitigating autoimmune responses.^3^ Indeed, IVIG has been demonstrated to benefit treatment of severe COVID-19.^4^ It is not too much of a stretch of the imagination to infer that a similar principle may be at play in the results indicated by Shrock et al.^1^ A further therapeutic application can also be observed in the apparent advantageous non-specific effects (NSEs) of live attenuated vaccines such as the Bacilli-Calmette-Guerin (BCG) and Polio (OPV) vaccines.^5^ The thought is that such vaccines broadly stimulate the innate immune system to confer non-specific protection against diseases other than the intended target of that vaccine, in essence ‘training’ components of the innate immune system.^5^ While the exact measurement of such protection provided remains unclear, numerous data suggest that at least some level of immunity is provided, with quantifiable reductions in mortality. Perhaps in such cases, a similar mechanism of prior ‘molecular immunity training’ is at play as observed by Shrock et al.^1^

Regardless, serological profiling has provided a window into viral responses amongst patients with diverse outcomes. In particular, we are only just beginning to understand how prior viral exposures may influence current/future responses. Of course, demographic and socioeconomic factors make it difficult to draw strong conclusions, and it will be some time before a suitable patient population can be examined to definitively obtain such answers. However, studies such as the one performed by Shrock et al. provide significant stepping-stones in understanding and isolating the molecular mechanisms of SARS-CoV-2 and COVID-19. Collectively, such milestones will potentially enable us to inform the production of improved diagnostics, therapeutics, and even vaccinations for SARS-CoV-2.^1^

## Supplementary information

Permission to use modified figure

## References

[1] Shrock E (2020). Viral epitope profiling of COVID-19 patients reveals cross-reactivity and correlates of severity. Science.

[2] Yaqinuddin A (2020). Cross-immunity between respiratory coronaviruses may limit COVID-19 fatalities. Med. Hypotheses.

[3] João C, Negi VS, Kazatchkine MD, Bayry J, Kaveri SV (2018). Passive serum therapy to immunomodulation by IVIG: a fascinating journey of antibodies. J. Immunol..

[4] Liu X, Cao W, Li T (2020). High-dose intravenous immunoglobulins in the treatment of severe acute viral pneumonia: the known mechanisms and clinical effects.. Front. Immunol..

[5] Chumakov K, Benn CS, Aaby P, Kottilil S, Gallo R (2020). Can existing live vaccines prevent COVID-19?. Science.

