# Supplementary figures and images for: COVID-19: cross-immunity of viral epitopes may influence severity of infection and immune response
Source: Signal Transduct Target Ther. 2021 Mar 1;6:102. doi: 10.1038/s41392-021-00490-x (PMC7919252; doi:10.1038/s41392-021-00490-x)

## Slide 1
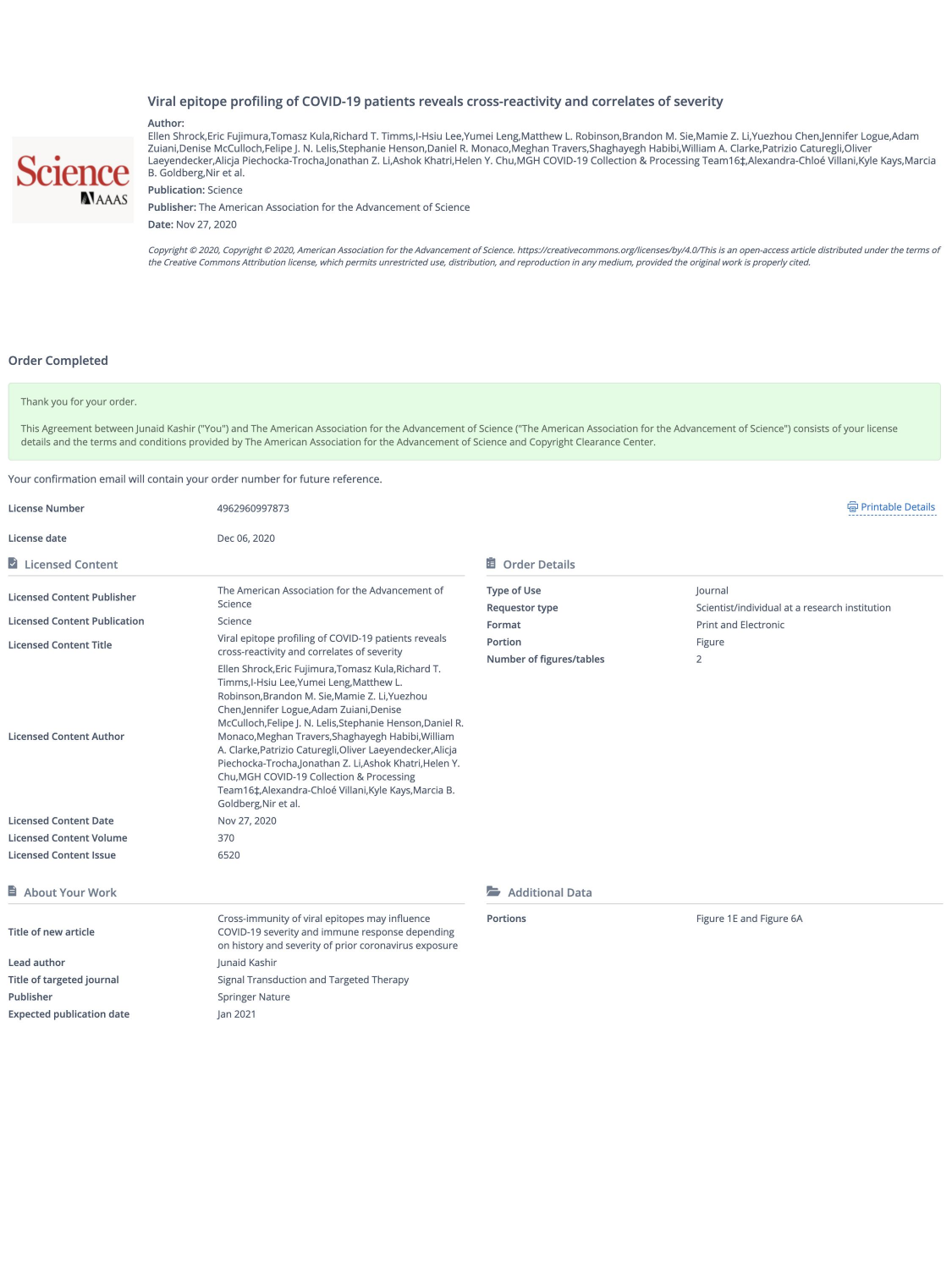

Supplement: Supplementary file 1 — Permission to use modified figure [file 41392_2021_490_MOESM1_ESM.pptx]
